# Mobile service robots for the operating room wing: balancing cost and performance by optimizing robotic fleet size and composition

**DOI:** 10.1007/s11548-022-02735-8

**Published:** 2022-09-11

**Authors:** Lukas Bernhard, Antony Francis Amalanesan, Oskar Baumann, Florian Rothmeyer, Yannic Hafner, Maximilian Berlet, Dirk Wilhelm, Alois Knoll

**Affiliations:** 1 Research Group MITI, Klinikum rechts der Isar, Technical University of Munich, Munich, Germany; 2 Chair of Robotics, Artificial Intelligence and Real-Time Systems, Technical University of Munich, Munich, Germany; 3 Chair of Materials Handling, Material Flow, Logistics, Technical University of Munich, Munich, Germany; 4 Department of Surgery, Klinikum rechts der Isar, Technical University of Munich, Munich, Germany

**Keywords:** Service robotics, Fleet management, Operating room wing, Surgical workflow optimization

## Abstract

**Purpose:**

Integrating fleets of mobile service robots into the operating room wing (OR wing) has the potential to help overcome staff shortages and reduce the amount of dull or unhealthy tasks for humans. However, the OR wing has been little studied in this regard and the requirements for realizing this vision have not yet been fully identified. This includes fundamental aspects such as fleet size and composition, which we have now studied comprehensively for the first time.

**Methods:**

Using simulation, 150 different scenarios with varying fleet compositions, robot speeds and workloads were studied for a setup based on a real-life OR wing. The simulation included battery recharging cycles and queueing due to shared resources.

**Results:**

For all simulated scenarios we report results regarding total duration of execution, average task response times and fleet utilization. The relationship between these performance measures and global scenario parameters—such as fleet size, fleet composition, robot velocity and the number of operating rooms to be served—is visualized.

**Conclusion:**

Our simulation-based studies have proven to be a valuable tool for individualized dimensioning of mobile robotic fleets, based on realistic workflows and environmental models. Thereby, important implications for future developments of mobile robots have been identified and a basis of decision-making regarding fleet size, fleet composition, robot capabilities and robot velocities can be provided. Due to costs, space limitations and safety requirements, these aspects must be carefully considered to successfully integrate mobile robotic technology into real-world OR wing environments.

## Purpose

Shortage of personnel is among the most pressing problems of modern healthcare systems around the world [1]. This issue is detrimental for various parts of the healthcare sector, including the management of operating room wings (OR wings), where a severe lack of qualified nurses and assistants has been observed for many years. As a result, fewer patients can be admitted for treatment and the main revenue center of surgical clinics becomes weakened. This challenge has been intensified by the prevailing COVID-19 pandemic [2], which necessitates complicated measures for surgically treating infected patients [3] and often requires the reassignment of surgical personnel and resources to support the overburdened wards and intensive care units.

Technologic means for overcoming the shortage of healthcare staff are widely explored in academia and industry and mainly draw from the fields of artificial intelligence, digitalization and robotics. This includes the application of mobile robotic assistance systems, which are very promising due to their high level of autonomy and the ability to traverse the environment. While mobile robotics has been explored for various use cases within the hospital [4–9], applications for the OR wing have not yet been studied comprehensively. At the same time, the potential for relieving humans from dull, repetitive or unhealthy tasks and for reducing the overall workload, is considerable. By means of robotic assistance, the human personnel could be relieved from tasks like the transportation of heavy objects—such as patient beds, medical devices, or instrumentation containers—or the cumbersome collection of packaged materials for upcoming surgeries, which is performed repeatedly throughout the day. Such an improvement of work ergonomics would be highly beneficial when considering the current working conditions of nurses [10] and help overcome today’s severe personnel shortage by making these jobs more attractive again.

Motivated by this vision, the current research project *Autonomous Self-Navigating Robotic OR Assistance* (AURORA) aims at designing and implementing a robotic circulating nurse for executing tasks within non-sterile areas of the OR wing. While a single such system operating independently can already be useful for supporting surgical interventions, the possibilities regarding workforce and intelligent workload distribution are limited. To use mobile robotic technology to its fullest potential, the formation of mobile robotic fleets for the OR wing is envisioned by the authors. This would allow for handling higher workloads and for implementing a context-dependent management of robotic resources, offering support where it is currently needed most.

Clearly, a successful clinical translation of this vision to real OR wing environments is not straight-forward and requires careful considerations regarding safety, hygiene and available space. One fundamental question is how large a robotic fleet should be to robustly handle the imposed workload, while limiting the required space and cost of operation to an acceptable level. We argue that this aspect is an important corner stone for demonstrating the general applicability of mobile robotics to OR environments and also sparks important requirements regarding the design of individual robots forming a fleet (e.g., concerning robotic capabilities and driving speeds). This includes the necessity of reaching performance levels comparable to human-only operation, or even surpassing it.

Similar investigations have been made by Jeon et al. regarding other hospital environments [11]. The authors present a simulation study analyzing a fleet of homogenous robots executing generic delivery tasks within the hospital. The aim of the study was to determine the optimal size of the fleet. However, the task plan to be executed by the robots was not based on actual recorded clinical workflows and the simulation model represents an office building environment, instead of an actual hospital. This raises concerns regarding the significance of the results since the requirements of different hospital processes and environments (ward, OR wing, emergency admission, laboratories, public spaces, etc.) are not taken into consideration.

To the best of our knowledge, the unique characteristics and requirements of the OR wing (e.g., regarding available space, safety requirements, reaction times, preparation phases vs. on-demand phases, types of tasks) have not yet been studied comprehensively in the context of mobile robotics. To bridge this knowledge gap and provide valuable insights for the AURORA project, we herein present the results of a simulation-based study and draw conclusions regarding optimal fleet sizes and compositions for a variety of different scenarios.

## Methods

### Simulation software

The results presented herein have been collected by means of simulation. Since commercially available or open-source tools failed to meet our specific requirements regarding flexibility and extensibility, we chose to implement a dedicated simulation software to be able to control every step of the process. Our ROS^1^- and Python^2^-based tool consists of modules for simulating the OR wing environment, the surgical workflow, and members of the mobile robotic fleet. Thus, it provides all puzzle pieces required for investigations regarding size and composition of mobile robotic fleets for the OR wing.

#### Environment

**Fig. 1:**
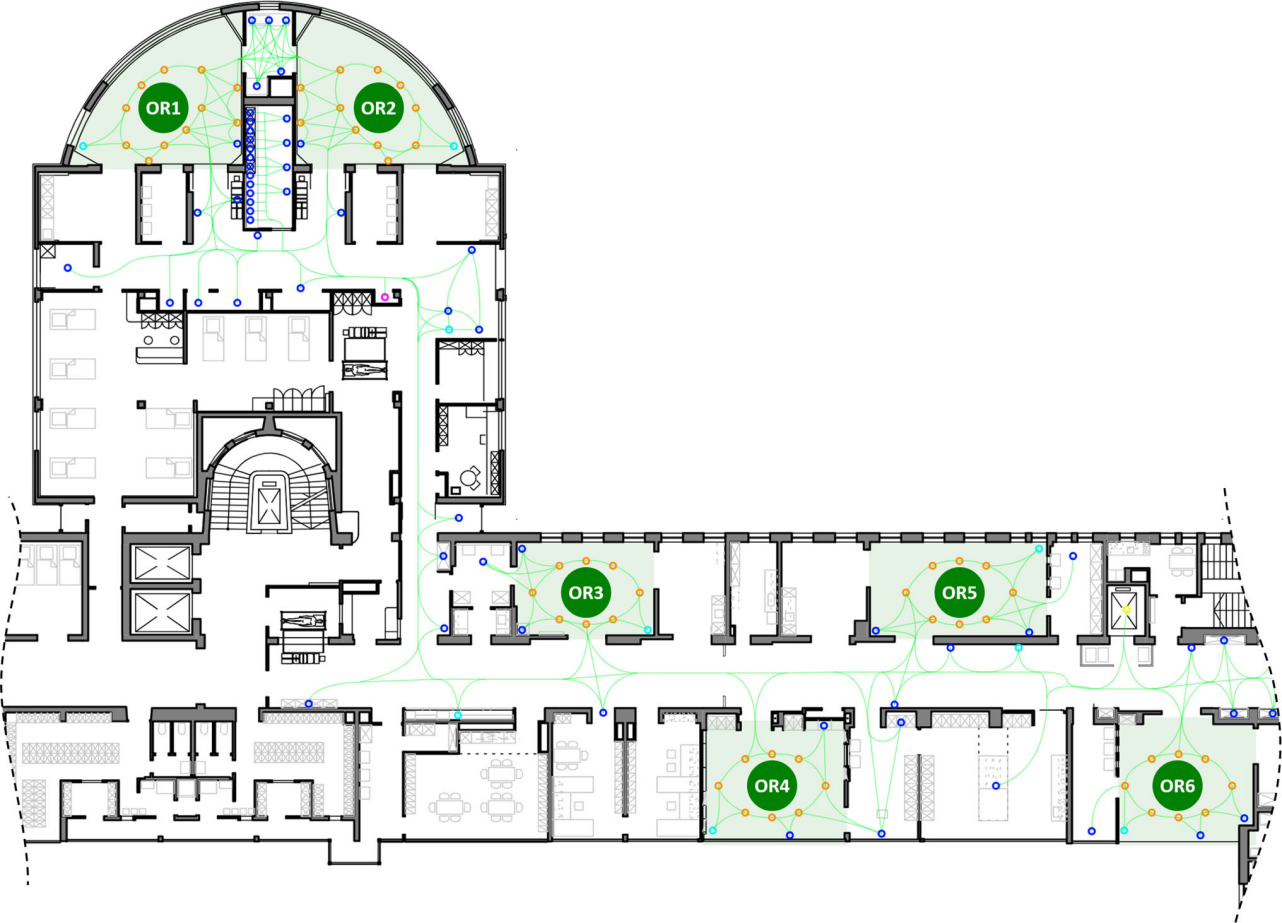
The architectural layout of the OR wing environment, as used by our simulation software, is shown. The environment model provides locations of robot bases, storages, tube mails, disposals, medical devices and other points of interest (*ring-shaped markers*), as well as possible driving paths for the robots (*green curves*)

The environment has been modeled closely to the real-life OR wing of a German university hospital. The layout is schematically shown in Fig. 1, including the size and location of eight operating rooms, and the location and inventory of storages. Further points of interest (POI), such as positions of medical devices, disposals, tube mail stations, etc., have been integrated into the environmental model as well.

#### Workflows

Prior to our simulation-based study, we have recorded the tasks of the circulating nurse during 20 laparoscopic cholecystectomies. We have selected this type of procedure since it is highly standardized (for a typical definition of workflow phases refer to [12]) and thus provides reliable data for the intended simulation, even with this comparatively low number of observations. The record includes preparation tasks that need to be carried out before the first incision as well as intra- and postoperative tasks. Intraoperative tasks are usually performed after having been requested by the surgical team, which is why we have recorded the time of request ${t}_{r}$, the time of execution start ${t}_{s}$ and the time of execution end ${t}_{e}$. Preparation and postoperative tasks, such as collecting the necessary instruments and sterile materials for an upcoming surgical intervention, are not performed on demand, but follow a loose routine prescribed by standard operating procedures. Here, it is not important to execute each individual task as soon as possible, but rather complete the entire collection of tasks until the scheduled beginning of the intervention (or the following intervention). For both the preparation process and the postoperative cleanup, we have recorded the collection of tasks carried out by human circulators and the timespan $\Delta {t}_{d}$ that was available for completing these tasks.

Using the workflow module of our simulation tool, an intervention schedule can be assembled for each operating room of our modeled environment based on recorded interventions. According to this schedule, the workflow module then simulates the workflow of the OR circulator by replaying the recorded data. This is achieved by spawning task requests according to the recorded request time and waiting until task execution has finished. The task requests are sent to the scheduler and then dispatched to the simulated robotic fleet for execution (also see section *Task Scheduling*). After task execution is finished, the workflow module is notified and continues to process the recorded surgery workflow.

Each task request contains a code defining the task type and a collection of additional type-specific parameters (e.g., desired objects and quantities). All different task types considered in our simulated scenarios are summarized in Table 1. Only such tasks of the circulating nurse were included that are suited for robotic automation. For the remaining types of tasks (e.g., documentation tasks or phone management), we argue that other solutions, such as digitalization, show greater potential.

**Table 1:** The different task types considered in our simulation study are described

Task type	Description
T1	Transportation of sterile goods (e.g., from storage to OR table)
T2	Transportation of a heavy load (e.g., containers, devices)
T3	Intra- or postoperative disposal of waste
T4	Delivery of lab samples to the OR wing’s tube mail station
T5	Adjustment of medical device
T6	Assistance during the sterile clothing procedure

For pre- and postoperative phases, the entire collections of tasks are requested at once and the workflow module monitors, whether all tasks are completed within $\Delta {t}_{d}$. If the execution is finished early by the robotic fleet, the workflow module waits for the remaining timespan, since $\Delta {t}_{d}$ is not only defined by the completion of circulator tasks but also by other external factors, such as patient transportation and anesthetic preparation. On the other hand, if $\Delta {t}_{d}$ is overrun, this is solely due to robotic task execution, and therefore all subsequent processes within the given OR are delayed.

#### Robotic Fleet

The fleet module of our simulation tool implements the behavior of different types of mobile robots and provides means for assembling a mobile robotic fleet based on these models. Table 2 summarizes all robot models implemented for simulating the scenarios presented in this paper.

**Table 2:** The different robot classes considered in our simulation study are described. Robotic capabilities are given with respect to the task types defined in Table 1

Robot class	Capabilities	Description
R1	T1, T3, T4	Transport robot for light loads
R2	T2	Transport robot for heavy loads
R3	T5	Robot for adjusting medical devices
R4	T6	Robot for assisting the sterile clothing procedure
R5	All (T1–T6)	All-rounder robot

Each simulated robotic fleet member provides an interface for receiving task requests dispatched by the scheduler. The execution of a task request is simulated by running a succession of the following three types of activities: driving, manipulating and waiting. For example, to simulate task type T1 (transportation of sterile goods), the robot first needs to navigate to the nearest storage containing the desired article (driving), then retrieve the article from the storage (manipulating), navigate to the operating table (driving) and hand over the article to the scrub nurse (manipulating). In case that one or multiple robots are currently blocking the storage or the hand-over position at the sterile table, the robot is enqueued and needs to wait for its turn (waiting). Driving is simulated by awaiting the travel duration, which is calculated based on the robot’s *driving speed* ${v}_{r}$ and the path length from robot position to target location. Manipulating is simulated by awaiting task-dependent durations that are specified within the robot model.

Since the battery capacity is an important factor limiting the operation of robotic resources, a battery simulation has been implemented as part of the robot models. While—depending on the power demand and battery capacity—overnight charging may suffice for some robotic systems, it may be a considerable limitation for others, especially when aiming at a lightweight robot design, which is preferable in the OR wing context. Therefore, we chose to include this aspect in our investigations.

During task execution, the robot’s battery is drained as follows, with $d$ being the drain rate, ${b}_{i}$ being the battery level at the current time step ${t}_{i}$, and ${b}_{i-1}$ being the battery level at the previous time step ${t}_{i-1}$:

$$ b_{i} = b_{i - 1} - d\left( {t_{i} - t_{i - 1} } \right) $$

To better reflect the individual power consumption associated with different parts of task execution, specific drain rates can be defined for driving (${d}_{d}$), performing (${d}_{p}$) and waiting (${d}_{w}$). If the battery level of a robot falls below a threshold ${b}_{low}$, the robot drives back to its base for recharging. The recharging process continues at least until the battery level ${b}_{high}$ is reached. Before that, the robot is not available for new task requests. In case that a robot has finished task execution and no new task is assigned for the moment, it navigates back to the nearest available base for recharging (regardless of its current battery level). The charging process is modelled accordingly using charging rate $c$:

$$ b_{i} = b_{i - 1} + c\left( {t_{i} - t_{i - 1} } \right) $$

### Task scheduling

In previous work, we have proposed the score-based context-adaptive scheduling algorithm *Auto-Navigation Task Scheduling for the Operating Room* (ANTS-OR) [13]. This scheduling algorithm considers context information associated with a given task (e.g., urgency due to emergency situations, command hierarchy) and prioritizes the task accordingly. While this approach is promising for managing mobile robotic fleets within the OR wing, it has not yet been fully evaluated in a structured manner, which is why we chose to use a basic “first-in-first-out” approach (FIFO) for our investigations regarding fleet size. While the FIFO algorithm is simple, it fits quite well with the “on-demand” style of task execution during surgical interventions, where new tasks are expected to be executed as soon as possible. FIFO scheduling also comes with the additional benefit of being computationally inexpensive, which is an important prerequisite for OR task scheduling, where short reaction times are usually expected or even strictly required in urgent situations.

The scheduling process is split into two parts: prioritization and dispatching. Prioritization is done by ranking all currently requested tasks with respect to their time of reception. Starting with the highest ranking task, the algorithm then dispatches the tasks to currently available fleet members. During this “match-making” process, the individual capabilities of the robots as well as their current state (position, battery level) are taken into account. Robots with a shorter traveling path during task execution are preferred over such with longer paths. Robots that are currently not needed for task executions or those with a battery level below ${b}_{low}$ are sent back to the nearest base.

### Simulated scenarios

In order to be able to make a statement regarding optimal fleet size, 150 different scenarios were simulated, which are summarized by Table 3. Each scenario represents a full day of OR wing operation with three surgical interventions being performed in each OR. Across the different scenarios, the types of robots constituting the fleet, the driving velocity of the robots, the number of ORs running in parallel, and the number of fleet members (i.e., the fleet size) were varied. The fleet was either composed of “all-rounder” robots, which are able to execute all considered types of task requests (robot class R5, see Table 2), or by “specialized” robots, which are only able to execute specific types of tasks (robot classes R1–R4). For the first case, all possible fleet sizes ranging from 1 to 20 members were simulated. For the latter case, the number of members was incremented from 1 to 5 for each robot class, resulting in fleet sizes of 4, 8, 12, 16 and 20 members. Regarding driving speeds, 0.3 and 1.2 m/s were studied, based on thresholds defined by DIN EN ISO 3691–4 (appendix A.2) [14]. Since the OR wing is a highly safety–critical environment (sterile zones, presence of humans, sensitive equipment, etc.), slow movements are preferred. At the same time, driving speed is an important limiting factor when it comes to the effectiveness of mobile robots and thus is relevant for determining optimal fleet sizes. Lastly, the number of operating rooms running in parallel is directly influencing the workload imposed on the robotic fleet. Thus, finding the optimal number of robots per OR is particularly relevant for balancing workforce and cost efficiency of the robotic fleet.

**Table 3:** 12 groups (G1–G12) of simulated scenarios are described based on robot types, driving speeds and the number of operating rooms used in parallel. For each group, fleet sizes between 1 and 20 robots (all-rounder robots) or, respectively, 4, 8, 12, 16 and 20 robots (specialized robots) have been studied

	G1	G2	G3	G4	G5	G6	G7	G8	G9	G10	G11	G12
Robot types	All-rounder robots (R5)						Specialized robots (R1–R4)					
Driving speed	0.3 m/s			1.2 m/s			0.3 m/s			1.2 m/s		
Number of ORs	2	4	6	2	4	6	2	4	6	2	4	6

The parameters of the battery simulation were set to constant values for all simulated scenarios and were identical for each robot class. Based on performance characteristics that we have identified as being favorable in the context of our AURORA robot, the lower battery level threshold was set to ${b}_{low}$ = 10%, the higher threshold to ${b}_{high}$= 50%. The drain rates were selected such that a full battery would allow for either 1 h of driving, 2 h of manipulating, or 4 h of waiting (or combinations thereof). The charging rate was defined such that a full charge is completed within 30 min (2C charging rate).

### Evaluation criteria

The aim of fleet size dimensioning is to keep operational costs as low as possible, while guaranteeing that the number of robots is high enough to sufficiently handle the workload imposed on the fleet. As soon as the fleet is no longer able to keep up with the demand, processing durations increase, which in turn leads to delays that are potentially affecting the entire OR wing operation.

One measure for quantifying this aspect is the *total duration* of surgical interventions, which ideally should not increase when compared to the human-only status quo. The elongation of surgical interventions results in increased costs (especially regarding personnel) and less time for treating subsequent patients, while the revenue per intervention remains constant.

From the perspectives of the surgical team and the patients currently undergoing surgery, *task response time* is another relevant measure, which refers to the timespan between task request ${t}_{r}$ and start of execution ${t}_{s}$. Intraoperatively, tasks are commonly requested by the surgical team “on demand”, and an immediate execution is desired. This is especially critical when it comes to emergency-related task requests, where the response time may be directly related to the wellbeing of the patient. In current clinical practice, short response times are commonly achieved by assigning at least one dedicated circulating nurse to each operating room.

Lastly, from an economic perspective, resources are usually meant to be used to their full capacity with as few down time as possible. This also translates to robotic fleet operation, where it is desired to avoid robots that are unoccupied (or in a fault state) for long time spans, while not creating any value. In the following, we quantify this aspect by defining the robot utilization ${\mu }_{r}$ as

$$ \mu_{r} = \frac{{t_{{{\text{active}}}} }}{{t_{{{\text{active}}}} + t_{{{\text{idle}}}} }} $$

using the active time ${t}_{active}$ (robot executes tasks) and the idle time ${t}_{idle}$ (robot charges or waits for new task assignment) of a given robot for a given simulated scenario. From this, the overall fleet utilization ${\mu }_{f}$ for a fleet composed of $n$ members can be calculated as the arithmetic average:

$$ \mu_{f} = \frac{1}{n}\mathop \sum \limits_{i = 1}^{n} \mu_{r,i} $$

## Results

**Fig. 2:**
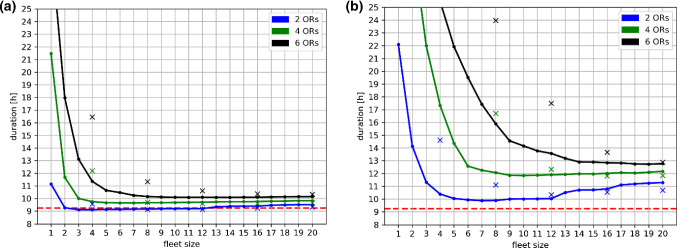
Total durations (in hours) are shown for all simulated scenarios. Part a) covers scenarios with robot velocities of ${v}_{r}=1.2 \,{\rm m/s}$, while part b) covers scenarios with ${v}_{r}=0.3 \,{\rm m/s}$. Durations for scenario groups G1–G6 (all-rounder robots) are depicted as continuous graphs, while durations for scenarios groups G7–G12 (specialized robots) are depicted as individual crosses. The number of operating rooms can be inferred from the color-coding defined by the figure legend. As a reference, the dashed red line defines the duration achieved for the human-only scenario, based on our recorded data

The total durations of all simulated scenarios are shown in Fig. 2. With increasing fleet size, durations decrease rapidly in an exponential-like fashion, until reaching a certain level of saturation. For scenario groups with two active operating rooms (scenario groups G1, G4, G7, G10), durations start to increase again after reaching a certain turnaround point (e.g., 12 robots for G1). In general, fleets consisting of all-rounder robots achieved shorter total durations compared to fleets consisting of specialized robots, except for fleet sizes greater than 12 for G1 and G2 as well as for fleet sizes greater than 8 for G4 and G5, where a slightly better performance can be observed. Notably, almost all scenarios resulted in a longer total duration than those achieved by the recorded human reference (1–2 dedicated circulating nurses per OR), with the exception of G4 for fleet sizes between 3 and 12 robots and G10 for fleet sizes of 8 and 12 robots.

**Fig. 3:**
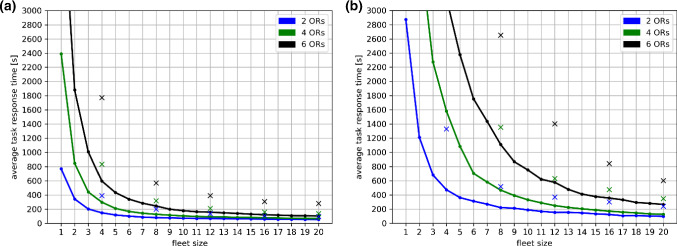
Average task response times (in seconds) are shown for all simulated scenarios. **a** ${v}_{r}=1.2 \,{\rm m/s}$. **b** ${v}_{r}=0.3 \,{\rm m/s}$

Average task response times are shown in Fig. 3. Again, times are decreasing for increasing fleet sizes until a level of saturation is reached. It can be observed that scenarios with all-rounder robots are consistently resulting in shorter response times than scenarios with specialized robots. Since the FIFO scheduling used in our simulations does not prioritize intra-operative tasks, we chose to present the average of all executed tasks, which includes preparation and post-operative tasks.

**Fig. 4:**
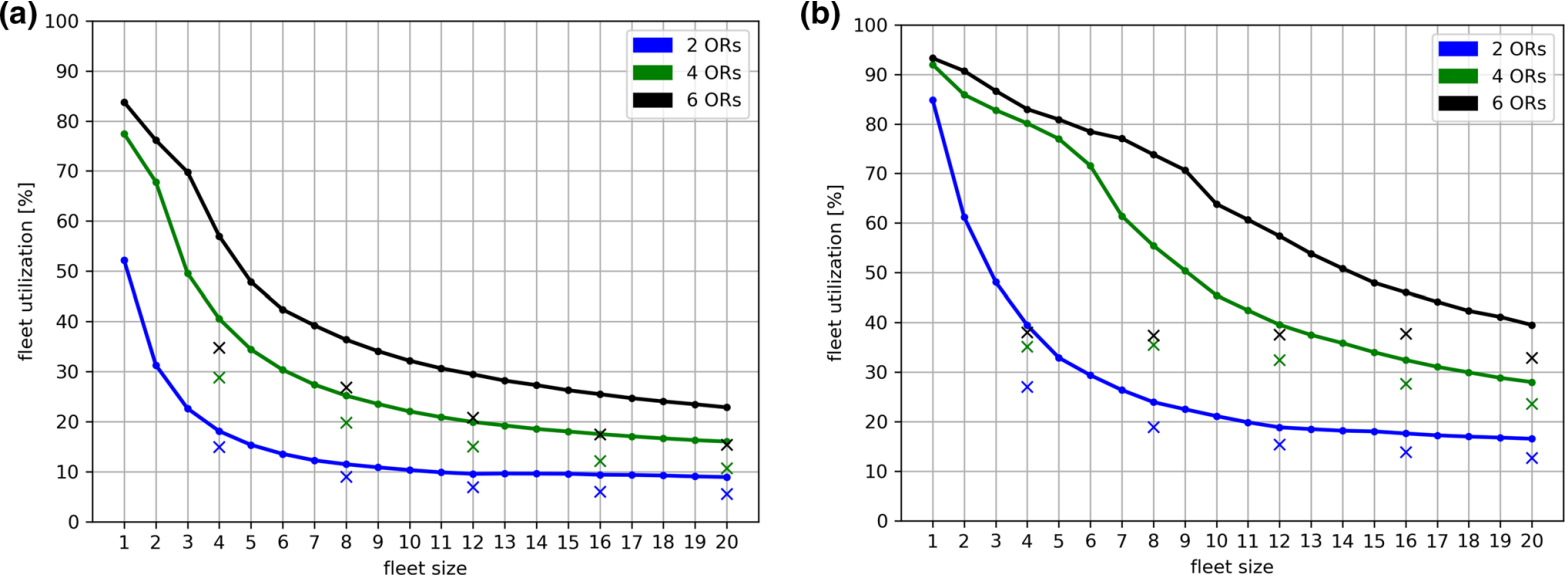
Fleet efficiencies (in percent) are shown for all simulated scenarios. **a** ${v}_{r}=1.2 \,{\rm m/s}$. **b** ${v}_{r}=0.3 \,{\rm m/s}$

Fleet efficiencies are shown in Fig. 4 for all simulated scenarios. As one would expect, utilization decreases for increasing fleet sizes, since the overall workload remains the same. For a high robot speed and a low number of ORs, an inverse-exponential behavior can be observed. For a slow robot speed and higher numbers of ORs, the graphs approach an inverse-linear behavior. Furthermore, it can be observed that scenarios with all-rounder robots are consistently resulting in higher fleet efficiencies than scenarios with specialized robots.

**Fig. 5:**
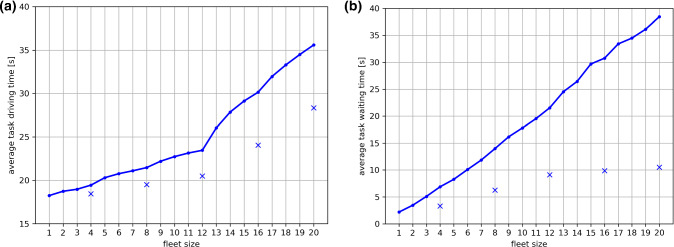
Average task driving times (part a) and average task waiting times (part b) are exemplarily shown for scenario groups G4 (continuous graph) and G10 (cross markers)

Figure 5 exemplarily shows average task driving and waiting times for scenario groups G4 and G10. As can be observed, both measures grow continuously for increasing fleet sizes. Scenarios with all-rounder robots consistently yield longer waiting and driving times than scenarios with specialized robots. For fleet sizes greater than 12 robots, waiting times start to increase at a faster rate than before. This behavior has not been observed in other scenario groups.

## Discussion

The results presented in the previous provide a valuable foundation for considerations regarding the dimensioning of mobile robotic fleets for the operating room wing. Based thereon, multiple conclusions can be drawn.

### Robot velocity

Our results demonstrate that robot velocity has a tremendous influence on all considered evaluation parameters. For example, task group G6 (6 ORs, ${v}_{r}=1.2 \,{\rm m/s}$) reaches its level of minimum total duration at approx. 10 h for a fleet size of approximately 8 robots and higher. Compared to that, task group G3 (6 ORs, ${v}_{r}=0.3 \,{\rm m/s}$) not only yields a minimum duration level that is approx. 27% higher, but also requires an increase in fleet size of 75% for reaching it. Similar observations can be made regarding average task response times. On the contrary, an increase in fleet utilization can be observed for lower robot velocities. However, this is only due to the entire system being overburdened and lagging behind, which results in a constantly high workload for the robotic fleet. While a high occupancy rate is usually desired and not unfavorable in and of itself, the tradeoff that has to be made regarding execution times is very high for the cases that we have investigated.

At the bottom line, this demonstrates that robot velocity is one of the key parameters when it comes to the feasibility of integrating mobile robotic fleets into the OR wing and should receive special consideration. Since the OR environment is highly safety–critical due to moving persons, sterile zones, sensitive equipment, etc., realistically achievable robot speeds are rather limited (e.g., with reference to common standards, such as [14]). While robot velocities of ${v}_{r}=1.2 \,{\rm m/s}$ have yielded promising results for our simulations, there might be considerable adaptations to today’s OR wing environments necessary to safely use such high velocities. One possible solution could be a “hybrid” approach, where robots are required to move slowly within the operating rooms, but are allowed to traverse hallways of the OR wing with a higher velocity, e.g., by implementing “robot-only” zones within the architectural layout. This could be realized by introducing virtual speed boundaries for the robots and may have to be complemented by modifications to the environment, such as dedicated and delimited driving lanes for robots. We plan to study the merits and limitations of this approach in future work.

### Fleet size

Our second conclusion is that enhancing the fleet size does not necessarily result in a better performance. While there is an obvious “natural” limit to fleet size due to the space required for driving and parking, other effects take over at an even earlier stage. After an increase of fleet size beyond a certain point, performance cannot be further improved. For scenarios with two operating rooms (G1, G4, G7, G10), we have even observed an inversion of total scenario durations. This effect can be explained with increased driving times, since the two ORs in the corresponding scenarios are located at one side of the OR wing (see OR 1 and 2 in Fig. 1) and bases close to these ORs fill up quickly. Thus, some robots are required to use bases that are more distant from the locations of actual task executions, which results in longer driving times, as supported by Fig. 5. For other scenarios, the locations of task execution are more spread out across the OR wing, and thus the probability of a robot being available at a base close to a given task request is higher.

Based on these results, one can infer recommendations regarding the optimal number of robots per OR. The goal is to find the smallest collection of robots that is still able to achieve an acceptable performance level. In the following, a total scenario duration that is at most 5% longer than the shortest total duration within the same scenario group (same number of ORs, same robot velocity) will still be considered acceptable. Regarding scenarios with all-rounder robots, this results in an optimal fleet size of 1 robot per OR for ${v}_{r}=1.2 \,{\rm m/s}$ (scenario groups G4–G6) and 2–3 robots per OR for ${v}_{r}=0.3 \,{\rm m/s}$ (scenario groups G1–G3). The use of specialized robots requires a larger fleet size even for scenarios with few operating rooms, resulting in 2 robots per OR for ${v}_{r}=1.2 \,{\rm m/s}$ (scenario groups G10–G12) and 3–6 robots per OR for ${v}_{r}=0.3 \,{\rm m/s}$ (scenario groups G7–G9). Exact numbers for each scenario group are given in Table 4.

**Table 4:** Based on the results of our simulations, recommended fleet sizes are given for all scenario groups

	G1	G2	G3	G4	G5	G6	G7	G8	G9	G10	G11	G12
Robot types	All-rounder robots (R5)						Specialized robots (R1–R4)					
Driving speed	0.3 m/s			1.2 m/s			0.3 m/s			1.2 m/s		
Number of ORs	2	4	6	2	4	6	2	4	6	2	4	6
Recommended fleet size	5	7	13	2	3	6	12	12	20	4	8	12

When considering the human performance as a reference (see red dashed line in Fig. 2), only few robotic scenarios achieve a better result. For slow robot velocities or more than two operating rooms, the total duration remains above human reference across all studied fleet sizes, which may be due to longer driving paths and queuing. Also, it must be pointed out that our workflow simulation is waiting in case all preoperative preparation tasks of a given intervention are finished earlier by the robots than by the recorded human reference ($\Delta {t}_{d}$, also see section *Workflows* in *Methods*). In this regard, our simulations are quite conservative.

One conceivable way to speed up robot-assisted workflows and possibly reach (or even surpass) human performance levels for more scenarios than currently observed would be the implementation of a task prediction algorithm (similar to surgical phase prediction [15]), which enables the robots to prepare likely tasks ahead of request. However, such a solution has not yet been presented in scientific literature, to the best of our knowledge.

### Fleet composition

Throughout the simulation results, a superior performance of all-rounder robots compared to specialized robots can be observed. For example, scenario group G6 (all-rounders, 6 ORs, ${v}_{r}=1.2 \,{\rm m/s}$) yielded an approx. 31% shorter total duration than scenario group G12 (specialists, 6 ORs, ${v}_{r}=1.2 \,{\rm m/s}$) for a fleet size of 4 robots. Likewise, fleet utilization is approx. 64% higher and response times are 66% shorter. While it is clearly to be expected that all-rounder robots lead to better performance levels with regard to these parameters, our results show that the gap between all-rounders and specialists is wide enough to make a significant difference regarding the practical feasibility of mobile robotic fleets for the OR wing. Thus, it can be concluded that the design of robots capable of executing more than a single task is highly beneficial from this point of view. Yet, this introduces challenges for robotic development, since realizing multiple capabilities within a single system usually leads to higher constructional complexity and manufacturing costs.

## Limitations

A limitation of our simulation-based study is that, as of yet, only one architectural layout has been considered. However, to ensure a high level of realism, the layout was closely modelled after the floorplan of an existing OR wing. In future work, we plan to investigate further existing and speculative layouts, which could be the foundation for formulating recommendations regarding optimal layouts for the integration of mobile robots. Furthermore, acceleration phases and collision avoidance between robots and obstacles were not considered by the simulation and would lead to elongated driving times in practice. It must also be mentioned that the recorded interventions were limited to cholecystectomies. Other types of interventions could potentially require the execution of more tasks per timespan and thus lead to a higher overall workload per OR that must be handled by the robotic fleet. This will also be further explored in future work.

Lastly, there is potential for increasing fleet performance by adopting approaches from related application domains, such as industrial intra-logistics. One example is smart vehicle routing, where robots execute multiple tasks along a planned route [16]. Thereby, driving durations can potentially be reduced.

## Conclusion

In this paper, we have presented a comprehensive simulation-based study regarding the dimensioning of mobile robotic fleets within the OR wing. We conclude that robot velocity is a crucial parameter when it comes to fleet performance and could be a potential hurdle regarding the integration of mobile robotic fleets into the OR wing. We further conclude that increasing the fleet size beyond a certain point does not necessarily lead to better results, especially when aiming for a good balance between fleet size and fleet performance. According recommendations regarding fleet size were given for the studied scenarios. Furthermore, we were able to confirm that a fleet consisting of all-rounder robots is advantageous regarding performance and utilization, as compared to a fleet of specialized robots. Therefore, it is desirable to design mobile robotic assistance systems that are capable of executing more than just a single specialized task—an important insight for future development efforts.

While some challenges have been identified, we consider our results to support the general feasibility of mobile robotic fleets for the OR wing. Depending on the studied parameters, scenarios have been identified that show an acceptable level of performance while requiring moderately sized robotic fleets.

## Footnotes

1 ROS Noetic Ninjemys (see https://wiki.ros.org/noetic).

2 Python 3.8.10 (see https://www.python.org/downloads/release/python-3810/).

## References

[1] Bär S, Starystach S (2017). Arbeitsbedingungen in der Krankenhauspflege. SozW.

[2] Cheeyandira A (2020) The effects of COVID-19 pandemic on the provision of urgent surgery: a perspective from the USA. J Surg Case Rep 2020(4):rjaa109. Doi: 10.1093/jscr/rjaa109 10.1093/jscr/rjaa109 PMC7176477 32346470

[3] Kibbe MR (2020). Surgery and COVID-19. JAMA.

[4] Scheidig A, Jaeschke B, Schuetz B, Trinh TQ, Vorndran A, Mayfarth A, Gross H-M (2019) May I keep an eye on your training? Gait assessment assisted by a mobile robot. In: IEEE international conference on rehabilitation robotics 2019:701–708. Doi: 10.1109/ICORR.2019.8779369 10.1109/ICORR.2019.8779369 31374713

[5] Ackerman E (2018) Moxi Prototype from diligent robotics starts helping out in hospitals. diligent robotics demos the latest version of their healthcare support robot. https://spectrum.ieee.org/moxi-prototype-from-diligent-robotics-starts-helping-out-in-hospitals

[6] Miseikis J, Caroni P, Duchamp P, Gasser A, Marko R, Miseikiene N, Zwilling F, de Castelbajac C, Eicher L, Fruh M, Fruh H (2020). Lio-A Personal robot assistant for human-robot interaction and care applications. IEEE Robot Autom Lett.

[7] Mamun KA, Sharma A, Hoque ASM, Szecsi T (2014 - 2014) Remote patient physical condition monitoring service module for iWARD hospital robots. In: Asia-pacific world congress on computer science and engineering. IEEE, pp 1–8

[8] Hasan MK, Hoque AS, Szecsi T Application of a plug-and-play guidance module for hospital robots. In: Proceedings of the 2010 international conference on industrial engineering and operations management, pp 654–659

[9] Bacik J, Durovsky F, Biros M, Kyslan K, Perdukova D, Padmanaban S (2017). Pathfinder-development of automated guided vehicle for hospital logistics. IEEE Access.

[10] Marć M, Bartosiewicz A, Burzyńska J, Chmiel Z, Januszewicz P (2019). A nursing shortage—a prospect of global and local policies. Int Nurs Rev.

[11] Jeon S, Lee J (2016) The simulator for performance analysis of multiple robots for hospital delivery. In: 2016 13th international conference on ubiquitous robots and ambient intelligence (URAI). IEEE, pp 746–748

[12] Twinanda AP, Shehata S, Mutter D, Marescaux J, Mathelin Md, Padoy N (2017). EndoNet: a deep architecture for recognition tasks on laparoscopic videos. IEEE Trans Med Imaging.

[13] Bernhard L, Ostler D, Feußner H, Wilhelm D (2020). Integrating autonomously navigating assistance systems into the clinic: guiding principles and the ANTS-OR approach. Int J Comput Assist Radiol Surg.

[14] DIN Deutsches Institut für Normung e. V (2020) Flurförderzeuge – Sicherheitstechnische Anforderungen und Verifizierung (Industrial trucks – Safety requirements and verification). Teil 4: Fahrerlose Flurförderzeuge und ihre Systeme (Part 4: Driverless industrial trucks and their systems)(DIN EN ISO 3691–4)

[15] Czempiel T, Paschali M, Ostler D, Kim ST, Busam B, Navab N, de Bruijne M, Cattin PC, Cotin S (2021). OperA: attention-regularized transformers for surgical phase recognition. Medical image computing and computer assisted intervention—MICCAI 2021.

[16] Shetty N, Sah B, Chung SH (2020) Route optimization for warehouse order picking operations via vehicle routing and simulation. SN Appl Sci 2(2). Doi: 10.1007/s42452-020-2076-x

